# Retraction: MiR-30a-5p Antisense Oligonucleotide Suppresses Glioma Cell Growth by Targeting SEPT7

**DOI:** 10.1371/journal.pone.0228340

**Published:** 2020-01-23

**Authors:** The PLOS ONE Editors

After this article [1] was published, a Correction was published [2] to address the following issues involving Fig 2:

- In Fig 2B1, the plots shown in SNB19 and LN229 control panels appeared similar in the original publication. LN229 Control and Scrambled panels were replaced in the updated version of this figure provided with the Correction.
- In Fig 2D1, there appeared to be areas of overlap between the images shown in SNB19 Control, Scrambled, and 30a-5p AS panels. The Control and Scrambled panels were replaced in the updated figure provided with the Correction.

Additional concerns were subsequently raised about results reported in Figs 3 and 5. Specifically:

- In Fig 3A, when levels are adjusted there appear to be horizontal and vertical lines or discontinuities around some cells, e.g. in the SNB19, 30a-5p AS CyclinD1 panel and the LN229, Control CyclinD1 panel. The corresponding author provided original images in support of these two panels and clarified that in preparing this figure they moved cell images from within the same original microscopy image, bringing more cells into the fields shown in the figure.
- There are similarities between the following data in Fig 3B and Fig 5:
  ○ Fig 3B, SNB19 β-actin lanes 2, 3 are similar to Fig 5, LN229 β-actin lanes 4, 3 when flipped horizontally.
  ○ Fig 3B, SNB19 SEPT7 lanes 2, 3 appear similar to Fig 5, LN229 SEPT7 lanes 4, 3, when flipped horizontally.
  ○ Fig 3B, LN229 MMP2 lanes 2, 3 appear similar to Fig 5, SNB19 MMP2 lanes 4, 3, when flipped horizontally.
  ○ Fig 3B LN229 MMP9, lanes 2, 3 appear similar to Fig 5, SNB19 CyclinD1 lanes 4, 3, when flipped horizontally.

In response to the western blot concerns, the authors noted that errors were made in preparing the β -actin panels. They provided a replacement image for Fig 3B SNB19 β -actin, and they provided western blot images cropped similarly to the published figure panels (although with additional lanes) to support several panels of Figs 3 and 5. No image data were provided to support the following, which include panels for which concerns were raised: Fig 3B, SNB19 cells, SEPT7, MMP2, MMP9; Fig 3B, LN229 cells, MMP9; and Fig 5, LN229 cells, Bcl2. The SNB19 PCNA image provided for Fig 3B differed from the published figure.

The original unadjusted and uncropped blot images supporting Figs 3 and 5 are not available. Without the original image data, we are unable to resolve the above concerns or verify the reported results.

In light of the above concerns, the *PLOS ONE* Editors retract this article.

In our discussion of this case, the authors clarified that in the Correction [2], the updated figure panels reported data from the original experiments. The original underlying data for Fig 2 are no longer available.

The authors either could not be reached or did not reply to the retraction notification.
